# Supplementary material for: Mechanistic Role of Jak3 in Obesity-Associated Cognitive Impairments
Source: Nutrients. 2022 Sep 9;14(18):3715. doi: 10.3390/nu14183715 (PMC9505565; doi:10.3390/nu14183715)
Supplement: Supplementary file 1 [file nutrients-14-03715-s001.zip › nutrients-1894393-SI.pdf]

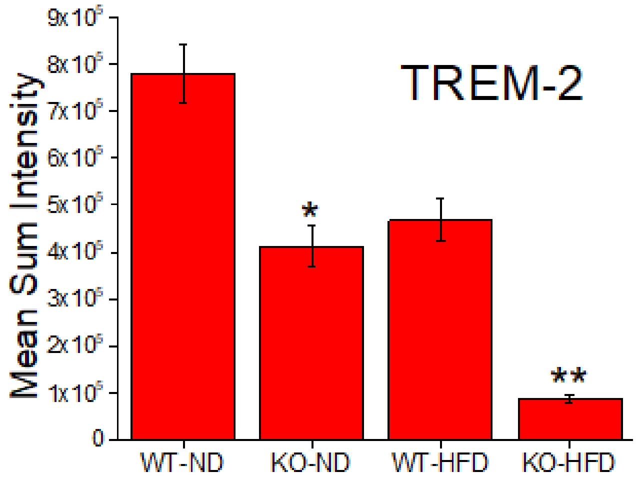

Supplemental **Figure S1:** Impact of global deficiency of Jak3 on the brain expression of β-Amyloid receptor TREM2. The quantification of the florescent pixels for TREM2 expressions in the brain from Figure 4C are shown. Data are representative of three independent experiments. Values are mean ± S.D. * denotes statistically significant compared with WT-ND mice and ** with WT-HFD mice (p > 0.01).

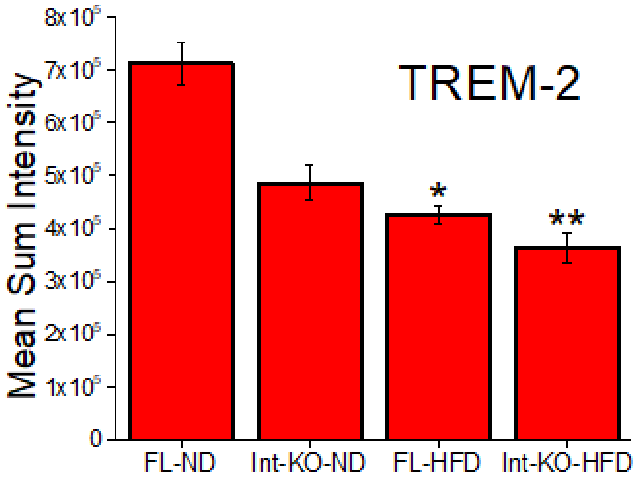

Supplemental **Figure S2:** Impact of IEC deficiency of Jak3 on the brain expression of β-Amyloid receptor TREM2. The quantification of the florescent pixels for TREM2 expressions in the brain from Figure 4E are shown. Data are representative of three independent experiments. Values are mean ± S.D. *, ** denotes statistically significant compared with FL-ND mice (p > 0.01).

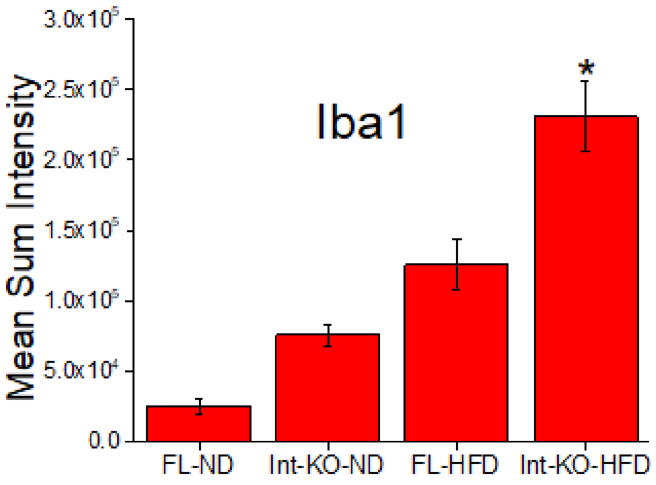

Supplemental **Figure S3:** Intestinal deficiency of Jak3 leads to increased microglial activation in the brain during HFD-induced obesity as determined through quantifications of the florescent pixels for microglial marker Iba1 from Fig 5C. Data are representative of three independent experiments. Values are mean ± S.D. *denotes statistically significant compared with FL-ND mice (*p* > 0.01).

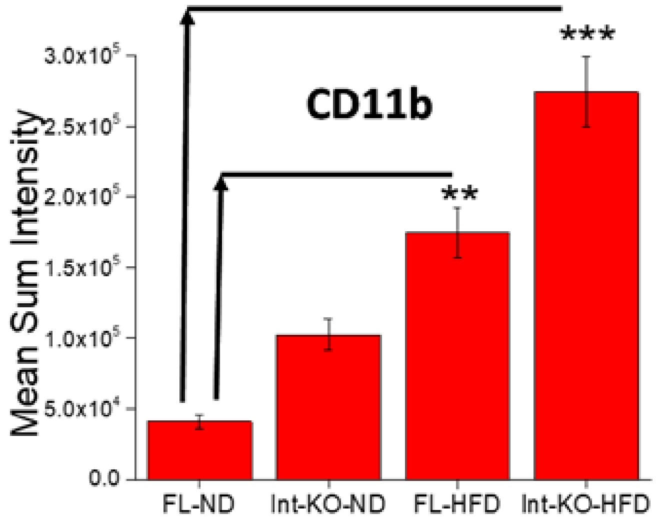

Supplemental **Figure S4:** Intestinal deficiency of Jak3 leads to increased microglial activation in the brain during HFD-induced obesity as determined through quantifications of the florescent pixels for microglial activation marker CD11b from Fig 5F. Data are representative of three independent experiments. Values are mean ± S.D. **, *** denotes statistically significant compared to the indicated groups (*p* > 0.01).

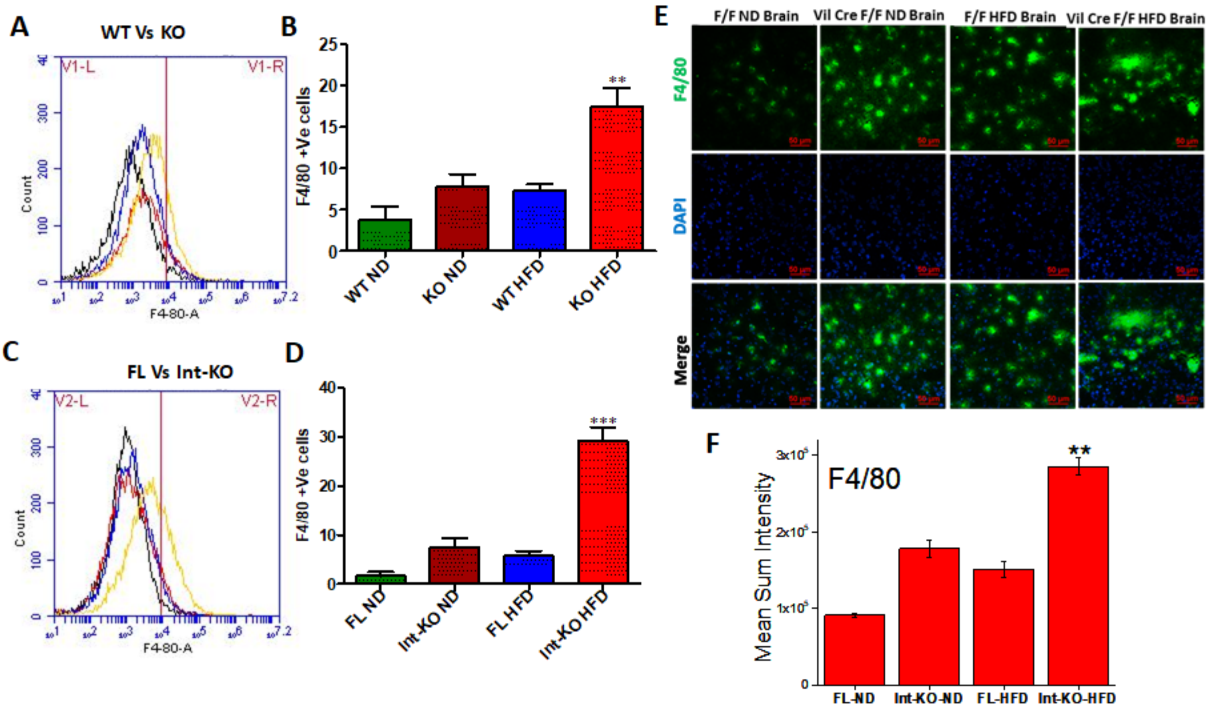

Supplemental **Figure S5**: Global or IEC deficiency of Jak3 is responsible for increased microglial activation in the brain as determined through F4/80. The impact of global (A,B) and intestinal epithelial tissue-specific (C-F) deficiencies of Jak3 on brain expression of microglial marker F4/80 was determined in ND- and HFD-fed mouse brain. Representative flow cytometric histogram graphs of individual mouse brain cells showing the microglial levels of expression of F4/80 in the four indicated groups of global deficiency or IEC deficiency, respectively (n = 5/group), are shown in the left panel, and the corresponding histogram bar graphs indicating mean ± SD values in the right panel are shown for the comparative average cell counts for the indicated groups of mice. **, *** Indicate statistically significant difference from the corresponding controls (*p* > 0.01). (E) Brain tissue sections from flox-Jak3-control littermate and IEC-Jak3-KO mice fed with either ND or HFD were immunostained using microglial marker F4/80 primary antibodies followed by FITC secondary antibodies, and mounting media containing PI were used to visualize the nucleus. Representative images (n = 10) are shown. (F) The quantification of the florescent pixels for F4/80 expressions in the brain from "E" are shown. Data are representative of three independent experiments. Values are mean ± S.D. ** denotes statistically significant compared with FL-ND mice (*p* > 0.01).
